# The influence of publication ranking specifications on publication strategy and academic careers in business administration

**DOI:** 10.1371/journal.pone.0336492

**Published:** 2025-11-25

**Authors:** Gerhard Reichmann, Christian Schlögl, Margit Sommersguter-Reichmann

**Affiliations:** 1 Department of Operations and Information Systems, Karl-Franzens University Graz, Graz, Austria; 2 Department of Finance, Karl-Franzens University Graz, Graz, Austria; Technical University of Aschaffenburg, GERMANY

## Abstract

This study examines the impact of methodological variations in publication-based rankings on the evaluation of individual research performance in business administration. Drawing on a unique dataset comprising complete personal publication lists of 233 professors from Austrian public universities (2009–2018), we apply ten distinct ranking variants that differ in their treatment of data sources, co-authorship, publication languages, article lengths, and journal qualities. These variants are categorized into purely quantity-focused and predominantly quality-focused rankings. Our results demonstrate that researcher rankings are susceptible to specification choices. While quantity-focused rankings produce relatively small performance differentials and high variability, quality-focused variants consistently identify a stable group of leading researchers. These scholars publish more frequently in English, in journals indexed by Web of Science (WoS), and in top-tier outlets according to the JOURQUAL ranking. Notably, leading researchers publish over twice as many articles in high-ranking journals as their peers. The findings underscore the significant implications of ranking design for career advancement and research strategy. For early-career researchers, aligning publication efforts with the logic of quality-focused rankings—favoring English-language publications in highly ranked, peer-reviewed journals—is crucial for enhancing academic visibility and competitiveness. Moreover, our study offers a methodological stress test for ranking systems, revealing the extent to which technical design influences outcomes. By leveraging comprehensive and multilingual publication data and systematically comparing multiple ranking methodologies, this study contributes to both the academic evaluation literature and practical guidance for researchers navigating the demands of a metric-driven academic environment.

## 1. Introduction

### 1.1. Background

Evaluation plays a crucial role in universities, influencing administration, teaching, and research. The academic literature reflects this, with studies evaluating administrative processes [1,2], teaching quality [3,4], and, in particular, research performance [5–7]. This paper focuses on research performance measurement as a core element of evaluation. Researchers assess research output at various levels, including universities [8], faculties or subject areas [9], departments [10], and individual scholars [11,12]. This study concentrates on the last group, individual researchers.

Research performance at the individual level can be measured in several ways, including publication and citation metrics, awards, prizes, editorial roles, and third-party funding [13–15]. We focus on publication output, which, along with citations, is the most widely used indicator in practice [16,17]. To ensure complete coverage, we use researchers’ personal publication lists rather than relying on external databases [18].

Multidisciplinary databases such as Web of Science (WoS), Scopus, or Google Scholar [19–21] often fail to capture non-English publications. This limitation is particularly significant for researchers in non-English-speaking regions. Meyer et al. [22] demonstrate that, in German-speaking business administration, the data source has a greater impact on ranking results than the use of publication counts versus citation counts.

Research rankings have evolved into more than mere documentation tools, as they increasingly influence career trajectories. High rankings signal achievement and offer career benefits, such as better job prospects or increased funding [23,24]. As a result, researchers have strong incentives to optimize their publication strategies.

Similar to other disciplines, rankings in business administration aim to identify ‘leading’ scholars at national or international levels. Most existing league tables rely on a single ‘best’ or most established variant. Yet a change in one specification, such as using fractional instead of full counting, can significantly shift ranking positions. Despite this, little is known about how such technical choices affect researcher rankings and the strategies behind them.

This study addresses this gap by systematically comparing multiple ranking variants and assessing their effects. We aim to:

- Test the stability of the top 10% of researchers (hereafter referred to as leading researchers) across various ranking variants.
- Identify the most influential publication characteristics (e.g., co-authorship, language, article length, or journal quality).

Our goal is twofold: (i) to offer evidence-based guidance to researchers, especially early-career scholars, on how publication strategies affect rankings, and (ii) to ‘stress test’ ranking systems by showing how sensitive they are to design choices. A key strength of our approach is the use of complete personal publication lists, which ensure full coverage and avoid the data gaps found in multidisciplinary databases, such as Web of Science (WoS) or Scopus.

This paper proceeds as follows: Section 1.2 reviews research rankings in the social sciences and economics, including those specific to German-speaking countries. Section 2 discusses the methods for individual and aggregate ranking variants, along with their practical relevance. Section 3 provides individual and aggregate ranking results, while Section 4 discusses the findings and the study's limitations. Section 5 concludes with a concise summary.

### 1.2. Literature overview

First, we searched the WoS for literature on publication rankings using the search term ‘publication-based rankings.’ Despite an unlimited period, the search yielded only a few publications focusing on ranking business administration units. Next, we expanded the subject area to encompass all social and economic sciences, ultimately identifying 46 relevant articles. As we found no studies covering business administration in German-speaking countries, we conducted another search outside WoS, yielding four additional papers.

#### 1.2.1. Search within the WoS.

Using the WoS search, we retrieved 46 papers that span the publication period from 1996 [25] to 2022 [26]. On average, each article covered ten years. The number of units analysed ranged from five to over 18,000, though most studies considered fewer than 100. Universities were the most frequently examined units. Other units included countries, schools, research programs, departments, and, as in our study, individual researchers.

Table 1 illustrates that 26 of the 46 studies (57%) restrict their analysis to a specific country or region, with the United States as the most frequent geographical focus. A single publication provided an economics ranking for Germany [27].

**Table 1:** Ranking studies retrieved from a WoS search (n = 46)).

Attributes	Specifications		
Geographical restrictions (n = 46)	No	*Yes*	
	(n = 20)	*(n = 26)*	
Discipline-specific restrictions (n = 46)	No	*Yes*	
	(n = 5)	*(n = 41)*	
Data sources (n = 46)	(Subject-specific) Journals	*WoS*	*Other*
	(n = 22)	*(n = 14)*	*(n = 10)*
Type of publications (n = 46)	*Journal articles*	Other	
	*(n = 41)*	(n = 5)	
Language restrictions (n = 46)	*No*	*Yes (English only)*	Other
	*(n = 44)*	*(n = 1)*	(n = 1)
Co-Authorship (n = 60*)	*Full Counting*	*Fractional counting (1/n)*	Other
	*(n = 33)*	*(n = 26)*	(n = 1)
Consideration of publication length (n = 46)	*No*	*Yes*	
	*(n = 37)*	*(n = 9)*	
Consideration of journal rankings (n = 46)	*No*	*Yes*	
	*(n = 26)*	*(n = 20)*	

Note: The specifications used in our study are written in *italics*.

*The 46 articles analyzed contained 60 ranking variants.

Nearly all (41 studies) limited their focus to a specific scientific discipline. Among these, 15 focused on economics (e.g., [28]), making it the most common field of study. Nine studies addressed business administration or its subfields, such as marketing [29], but none specifically focused on German-speaking countries.

The dominant data sources are (subject-specific) journal lists, used in 22 studies, followed by WoS (14), and other sources (10). The number of journals used per study ranges from 2 to 258. Scopus and Google Scholar appear only twice each; only one study used personal publication lists, likely due to the considerable effort required to compile them. However, personal publication lists generally provide more complete and higher-quality data.

Journal articles are by far the most common publication type assessed (41 of 46 studies), reflecting standard practice in most disciplines [6,30].

While scholars critically view language restrictions, especially English-only criteria [31], such filters are rarely applied explicitly: 44 studies accepted all languages, with only one limited to English and another to Chinese. Nonetheless, the reliance on multidisciplinary databases like WoS or Scopus implicitly narrows the scope to English-language publications, as these sources predominantly index English-language journals. In one variant of our study, we limited the sample to English-language articles to explore how results differ when relying solely on WoS instead of personal publication lists. Substantial differences suggest that even English-language articles from our authors are not indexed there.

Across the 46 studies, we identified 60 distinct ranking variants, exceeding the number of articles due to different approaches to considering multiple authorship. More than half of the studies employed full counting (33) over fractional counting (27 studies, with 26 using the formula 1/n, with n representing the number of authors), despite full counting being considered unfair due to the disadvantage it poses for single authors [32–34]. Reflecting this concern, eight out of ten variants in our study apply fractional counting.

Only nine of the 46 articles adjusted for publication length, typically using unstandardized page counts, while 37 did not (e.g., [35,36]). Similarly, only 20 studies applied journal-ranking weights. These weights reflect a journal's value, based either on subjective, discipline-specific rankings (e.g., JOURQUAL; [37]) or objective metrics, such as the journal impact factor (IF) [38,39]. Among the 20 studies, WoS-IF was the most frequently used (7 cases), followed by various subject-specific rankings, especially in economics.

Overall, discipline-specific rankings of journal articles, often compiled from bespoke journal lists, dominate the literature. Language restrictions are rare, while studies paid limited attention to publication length or journal quality metrics.

#### 1.2.2. Search outside the WoS.

To capture potential discipline- and region-specific aspects, we supplemented the WoS search with a review of business administration studies from the German-speaking region, resulting in four additional papers (Table 2). Studies ranking individual researchers in this context are scarce. Meyer et al. [22] evaluated the research performance of 298 accounting and marketing scholars in German-speaking countries, utilizing WoS, Scopus, Google Scholar, and Handelsblatt as data sources. These sources yielded substantial variation in publication counts due to differences in completeness levels. The authors applied full counting for co-authorship and measured journal quality in one case using the Handelsblatt ranking [40,41]. The findings highlight the substantial impact of the data source on individual rankings. Our study addresses these issues by utilizing personal publication lists, which provide greater completeness, taking into account fractional counting and different variants in journal quality.

**Table 2:** Ranking studies retrieved from a search outside the WoS (n = 4)*.

	Meyer et al., 2012 [22]	Fülbier and Weller, 2011 [42]	Rost and Frey, 2011 [43]	Macharzina et al., 2004 [44]
Geography	Germany, Austria, Switzerland	Germany	No restriction	Germany, Austria, Switzerland
Discipline	Accounting and Marketing	Financial Accounting	Organization Management	Business Administration
Data sources	WoS, Scopus, Google Scholar, Handelsblatt	JOURQUAL A + , A-, and B-ranked journals	11 high-quality journals	6 major German-language journals
Publication types	No restriction	Journal articles	Journal articles	Journal articles
Publication language	No restriction	No restriction	No restriction	German only
Co-Authorship	Full Counting	Full Counting	Fractional Counting	Fractional Counting
Publication Length	No consideration	No consideration	Number of pages	No consideration
Journal quality	Partly	JOURQUAL	Various rankings	No consideration

*Limited to business administration and/or the German-speaking region and to publication-based ranking variants

Fülbier and Weller [42] analysed 175 German financial accounting researchers between 1950 and 2005, using journals ranked at least JOURQUAL B. They identified 733 articles, with approximately 87% published in German. Using full counting, they found that 20 leading researchers (11%) accounted for 28% of all publications. We avoid two limitations of their approach: exclusive reliance on full counting and the restriction to discipline-specific journals, which can disadvantage both single authors and interdisciplinary researchers.

Rost and Frey [43] examined the relationship between quantitative (publications, citations) and qualitative (editorial board memberships) performance indicators for 851 management scholars. They used 11 high-quality journals, selected based on various rankings, over a ten-year period. Rankings were based on raw and weighted publication counts, accounting for article length, journal IF, and co-authorship through fractional counting. While exploring multiple ranking variants, the authors relied on a narrow database of just 11 discipline-specific journals, which likely facilitated broader researcher inclusion but limited generalisability.

Macharzina et al. [44] ranked researchers and practitioners based on their publications in six major German-language business journals, identifying 2,255 articles over ten years. They used fractional counting (1/n) and included 142 individuals with at least three research points (the latter represent the number of publications utilizing the formula 1/n for co-authorship; in our study, however, we retain the term ‘publications’ as measurement unit for this case), comprising 105 professors, 16 junior researchers, and 21 practitioners. However, the study is over 20 years old, relies exclusively on German-language journals, and omits international outlets—an increasingly important venue for German-speaking researchers. In contrast, our study ensures a more homogeneous sample (professors only) and includes both German- and English-language publications.

#### 1.2.3. Synthesis gaps.

Table 3 contrasts six recurring shortcomings in previous researcher-ranking studies with the approach taken in our research to address these shortcomings. Although many studies zero in on a single discipline or country, none cover Austrian business economists; moreover, most still rely on incomplete databases (WoS, Scopus, Google Scholar, or narrow journal lists), overlook language bias, credit every co-author fully, build on tiny sets of field journals, and analyse datasets that are now decades old. Our study addresses each of these issues by examining the publication records of business administration professors working in Austria, utilizing complete personal publication lists, conducting language sensitivity checks, applying mainly fractional counting, and covering output through 2018.

**Table 3:** Synthesized gaps from prior works.

Attributes	Prior works	Our study
Discipline & region coverage	Only one of 46 WoS ranking studies covers the German-speaking area, and that in economics, not business administration [27].	We analyze business administration scholars working in Austrian public universities.
Data source completeness	Almost all of the 50 analyzed studies draw on WoS, Scopus, or ad-hoc journal lists; personal publication lists, arguably the most complete source, were used only once [64].	We compile complete publication lists for every researcher, reducing database omissions.
Language bias	Of 46 WoS-based studies, only two report language restrictions [56,65].	We run parallel variants with (i) all languages and (ii) English-only publications to quantify WoS's language bias.
Counting method for co-authorship	Thirty-three variants in the 46 WoS studies use full counting, while 27 apply fractional counting (mostly 1/n). Of the four relevant articles outside the WoS, two use full counting while the other two use fractional counting.	Eight of our ten variants apply fractional counting (1/n) to avoid penalizing single authorships.
Small journal sets	Several influential rankings rely on no more than 11 discipline-specific journals (e.g., [43]).	Our dataset is source-agnostic and therefore captures cross-disciplinary and non-listed outlets.
Out-of-date evidence	The most comprehensive German-language ranking [44] is over two decades old and confined to German-language journals.	We update the evidence base with data through 2018 and include multilingual output.

These gaps motivate our central aims: (a) test the stability of ‘top-10%’ researcher status across ten distinct ranking variants, and (b) identify publication attributes (e.g., co-authorship, language, length, journal quality) that drive rank shifts. By doing so, we provide actionable guidance to early-career scholars and a methodological’ stress-test ‘for ranking producers.

## 2. Materials and methods

This study focuses on researchers with professorial titles (full, associate, or assistant professors) at Austrian public universities offering Bachelor's or Master's programs in business administration. We further limited the sample to individuals affiliated with faculties of social and economic sciences, primarily in business administration departments or related chairs. We justify this approach based on the homogeneity of the scholars. We define homogeneity as follows:

- Academic comparability: All researchers hold a professorial title, ensuring similar levels of education, research experience, and academic independence.
- Organizational focus: All scholars work in units with a clear business administration orientation, regardless of individual educational or research backgrounds.

To facilitate a comprehensive comparison, we limited the sample to Austria as encompassing the entire German-speaking region would have exceeded our available resources. We further limited the scope to public universities to ensure comparable institutional conditions. However, this had no practical effect, as no private universities in Austria offered relevant programs at the time. We did not consider researchers from economics and sociology. As separate disciplines with distinct degree programs, economics has its own established rankings, and sociology follows a different publication culture, with a strong focus on monographs. However, we included staff from operations research and business informatics when their organizational assignment placed them within a Business School.

We only included researchers who (a) held a permanent university position, (b) earned a doctoral degree before 2009, and (c) were employed at one of six designated Austrian public universities as of July 31, 2019. Based on university websites, we identified 233 researchers who met all criteria. For these 233 individuals, we collected all journal articles (research and review papers) published between 2009 and 2018. The ten-year window and the focus on journal articles align with standard practices in researcher-level assessments. In business administration, journal articles carry more weight for career advancement than monographs, proceedings, or other types of publications [16,44]. This preference is partly due to the challenges of objectively assessing non-journal outputs [45]. We compiled the data between March and June 2020, using the researchers’ personal publication lists, which yielded 4,246 journal articles. Personal publication lists are considered the most complete data source, essentially the gold standard, because they typically include all publications, not just those published in journals covered by the bibliometric databases. Most Austrian public universities require researchers to maintain these records through internal databases, which can automatically generate personal publication lists.

In addition to standard bibliographic information, we recorded i) the number of authors, ii) language (German or English), iii) number of pages, and iv) journal coverage in WoS for each relevant article.

To assess journal quality, we documented the JOURQUAL-3 rating and 2-year WoS-IF as of summer 2023. JOURQUAL-3 is the standard in German-speaking business administration in its third edition, while WoS-IF is an international standard across disciplines.

Methodological choices made before evaluation can significantly influence ranking outcomes [46]. Despite this, most studies apply only one preferred ranking method [26,47,48]. These methods vary in terms of data source, treatment of co-authorship, publication language, and journal weighting.

Based on our literature review (see Section 1.2), we selected ten ranking variants (V1–V10) to assess the 233 researchers. Table 4 summarizes their specifications regarding data source, co-authorship, language, length, and journal quality.

**Table 4:** Ranking variants of our study.

		Category 1				Category 2					
**Attributes**	**Specifications**	V1	V2	V3	V4	V5	V6	V7	V8	V9	V10
											
Data Sources	Publication List	x	x	x	x	x		x	x	x	x
	WoS						x				
Co-Authorship	Full Counting	x								x	
	Fractional Counting (1/n)		x	x	x	x	x	x	x		x
Publication Language	All Languages	x	x	x	x		x	x	x	x	x
	English Only					x					
Publication Length	Not relevant	x	x			x	x	x	x	x	x
	Page Number			x							
	5 Pages Lower Limit				x						
Journal Ranking	Not relevant	x	x	x	x	x	x				
	WoS Impact Factor							x			
	JOURQUAL A + , A, and B								x		
	JOURQUAL A+ and A									x	x
Measurement Unit	Publications	x	x		x	x	x		x	x	x
	Publication Points							x			
	Pages			x							

Note: All variants except V3 and V7 use *publications* as the unit of measurement. Variant V3 utilizes pages to accommodate publication length. Variant V7 applies *publication points*, calculated by multiplying each article by the journal's WoS-IF at the time of publication.

We also grouped the rankings into Category 1 and Category 2 rankings. The key distinctions lie in 1) whether the ranking is quality-focused or quantity-focused, i.e., whether the ranking explicitly considers journal quality, typically operationalized through journal rankings; and 2) whether the practical relevance is high. We assigned rankings V1 to V5 to Category 1 and the remaining variants V6 to V10 to Category 2. While V7-V10 do incorporate journal rankings, V6 does not take journal rankings into account. Nevertheless, we assigned V6 to Category 2, as it is widely used as a de facto standard in evaluation practice [5], despite not accounting for journal quality. In this sense, V6 functions similarly to quality-focused rankings by shaping research evaluation and academic careers.

Table 4 shows that we applied fractional counting (1/n) in eight cases. We also considered full counting in two variants (V1, V9) to assess its effect despite being outdated [22,42]. To investigate the impact of language filters, V5 considered English-only publications. Regarding publication length, V3 used page counts, whereas V4 employed a minimum of five pages [6,44]. We incorporated journal quality in four Category-2 rankings (V7–V10). This approach reflects the pivotal role of journal rankings in hiring decisions and academic careers. The literature review reports similar findings, where 20 of 46 studies utilized journal rankings, most often subject-specific, survey-based lists. We used JOURQUAL in three variants (V8–V10), filtering for A + , A, and B journals only [42,49]. In V7, we weighted articles using the WoS-IF. We excluded articles without any WoS-IF, highlighting our focus on consistent quality standards.

We then applied the ten variants to rank the 233 researchers. We focused on the top 10%, i.e., the so-called leading researchers (23 individuals; see Section 3.1) and analyzed which attributes exerted the highest influence on the top positions.

To check robustness, we created two aggregate rankings (see Section 3.2):

- The first includes all researchers ranked in the top 10% in at least one of the ten variants.
- The second incorporates only those ranked in the top 10% in at least one of the five Category-2 variants (V6–V10).

Finally, we identified the attributes that most strongly contributed to being among the leading researchers.

## 3. Results

### 3.1. Leading researchers: individual rankings

Table 5 presents the ten ranking variants detailed in Table 4, highlighting the upper echelon of researchers, specifically, the 23 leading researchers of the 233 evaluated. Each ranking builds on column three, which varies to include publications (V1, V2, V4, V5, V6, V8, V9, and V10), publication points (V7), or number of pages (V3), depending on the variant. For fractional counting (V2, V3, V4, V5, V6, V7, V8, and V10), we applied the formula 1/n.

**Table 5:** Leading researchers: individual rankings V1-V10.

V1 (Publication Lists, Full Counting)										V2 (Publication Lists, Fractional Counting)								
Name	Rank	Publications	Co-Authored Publications (%)		Publications in English (%)	Average Number of Pages	WoS-IF Publications (%)	A + /A/B JOURQUAL Publications (%)	A + /A JOURQUAL Publications (%)	Name	Rank	Publications	Co-Authored Publications (%)	Publications in English (%)	Average Number of Pages	WoS-IF Publications (%)	A + /A/B JOURQUAL Publications (%)	A + /A JOURQUAL Publications (%)
P151	1	166	93		–	4.4	–	–	–	P151	1	78.7	93	–	4.4	–	–	–
P210	2	156	100		–	1.4	–	–	–	P210	2	77.7	100	–	1.4	–	–	–
P153	3	98	94		–	2.6	–	–	–	P153	3	51.7	94	–	2.6	–	–	–
P187	4	82	99		100	17.6	21	44	2	P063	4	48.3	58	1	6.0	–	–	–
P135	4	82	100		100	16.6	73	68	41	P086	5	33.3	88	22	11.0	4	7	1
P063	6	69	58		1	6.0	–	–	–	P082	6	29.7	79	–	11.7	–	–	–
P086	7	67	88		22	11.0	4	7	1	P156	7	28.7	88	–	4.6	–	–	–
P106	8	64	94		81	16.5	42	44	11	P081	8	25.4	88	–	12.3	–	–	–
P082	9	63	79		–	11.7	–	–	–	P088	9	25	74	67	20.9	16	9	–
P081	10	60	88		–	12.3	–	–	–	P135	10	24.8	100	100	16.6	73	68	41
P049	11	58	100		95	14.6	50	40	12	P233	11	24.3	41	18	4.4	3	–	–
P123	12	55	95		100	15.5	82	82	25	P187	12	24.3	99	100	17.6	21	44	2
P156	13	52	88		–	4.6	–	–	–	P123	13	23.7	95	100	15.5	82	82	25
P113	13	52	100		100	23.4	–	8	–	P106	14	23.3	94	81	16.5	42	44	11
P090	15	51	98		57	16.6	10	16	–	P124	15	21.2	83	86	10.0	33	31	–
P054	16	47	100		91	17.0	9	6	–	P109	16	20.7	76	84	17.0	5	8	–
P171	16	47	96		100	16.4	11	9	–	P099	17	20.6	89	44	15.5	24	29	–
P146	18	45	98		100	18.6	40	31	13	P148	18	19.5	42	100	11.0	8	8	–
P136	18	45	100		100	20.0	71	69	38	P093	19	19.4	43	100	7.0	–	–	–
P099	18	45	89		44	15.5	24	29	–	P103	20	19.3	83	3	6.3	–	3	–
P206	21	44	98		98	15.9	70	64	5	P090	21	18.8	98	57	16.6	10	16	–
P088	22	43	74		67	20.9	16	9	–	P146	22	18.6	98	100	18.6	40	31	13
P124	23	42	83		86	10.0	33	31	–	P171	23	17.9	96	100	16.4	11	9	–
Mean value per										Mean value per								
Leading Researcher (n = 23)		67	92		58	13.4	24	24	7	Leading Researcher (n = 23)		30.2	83	51	11.5	16	17	4
Non-Leading Researcher (n = 198)		14	85		76	16.1	31	32	11	Non-Leading Researcher (n = 198)		6.1	86	77	16.4	32	33	11
**V3 (Publication Lists, Fractional Counting, Publication Length: Page Numbers)**											**V4 (Publication Lists, Fractional Counting, Publication Length: 5 Pages Minimum)**							
**Name**	**Rank**	**Pages**	**Co-Authored Publications (%)**		**Publications in English (%)**	**Average Number of Pages**	**WoS-IF Publications (%)**	**A + /A/B JOURQUAL Publications (%)**	**A + /A JOURQUAL Publications (%)**	**Name**	**Rank**	**Publications**	**Co-Authored Publications (%)**	**Publications in English (%)**	**Average Number of Pages**	**WoS-IF Publications (%)**	**A + /A/B JOURQUAL Publications (%)**	**A + /A JOURQUAL Publications (%)**
P088	1	498.2	74		67	20.9	16	9	–	P063	1	31.2	58	1	6.0	–	–	–
P187	2	413.9	99		100	17.6	21	44	2	P151	2	27.8	93	–	4.4	–	–	–
P135	3	400.8	100		100	16.6	73	68	41	P187	3	23.6	99	100	17.6	21	44	2
P086	4	362.0	88		22	11.0	4	7	1	P135	4	23.2	100	100	16.6	73	68	41
P146	5	350.7	98		100	18.6	40	31	13	P088	5	22.6	74	67	20.9	16	9	–
P123	6	348.0	95		100	15.5	82	82	25	P086	6	22.3	88	22	11.0	4	7	1
P109	7	346.8	76		84	17.0	5	8	–	P123	7	20.3	95	100	15.5	82	82	25
P106	8	343.5	94		81	16.5	42	44	11	P106	8	19.5	94	81	16.5	42	44	11
P041	9	334.6	86		75	23.3	32	57	14	P109	9	19.1	76	84	17.0	5	8	–
P151	10	333.9	93		–	4.4	–	–	–	P146	10	18.3	98	100	18.6	40	31	13
P043	11	329.2	79		58	25.5	21	33	13	P099	11	17.8	89	44	15.5	24	29	–
P099	12	298.2	89		44	15.5	24	29	–	P148	12	17.5	42	100	11.0	8	8	–
P090	13	295.7	98		57	16.6	10	16	–	P171	13	17.4	96	100	16.4	11	9	–
P054	14	293.0	100		91	17.0	9	6	–	P054	14	17.3	100	91	17.0	9	6	–
P063	15	288.2	58		1	6.0	–	–	–	P206	15	17.0	98	98	15.9	70	64	5
P171	16	283.7	96		100	16.4	11	9	–	P090	16	16.6	98	57	16.6	10	16	–
P082	17	283.1	79		–	11.7	–	–	–	P049	17	16.5	100	95	14.6	50	40	12
P112	18	278.5	90		60	17.4	23	23	10	P143	18	16.1	86	100	17.4	67	31	28
P143	19	278.2	86		100	17.4	67	31	28	P082	19	15.5	79	–	11.7	–	–	–
P081	20	277.8	88		–	12.3	–	–	–	P011	20	15.0	61	17	10.5	–	9	–
P136	21	267.8	100		100	20.0	71	69	38	P081	21	14.7	88	–	12.3	–	–	–
P113	22	267.6	100		100	23.4	–	8	–	P024	22	14.2	62	81	17.1	57	67	33
P028	23	260.3	87		39	17.2	23	45	10	P065	23	14.0	65	–	7.0	4	–	–
Mean value per										Mean value per								
Leading Researcher (n = 23)		323.2	89		64	16.4	25	27	9	Leading Researcher (n = 23)		19.0	84	63	14.2	26	25	8
Non-Leading Researcher (n = 198)		91.0	86		75	15.8	31	31	11	Non-Leading Researcher (n = 198)		5.2	86	75	16.0	31	32	11
**V5 (Publication Lists, Fractional Counting, English only)**										**V6 (WoS, Fractional Counting)**								
Name	Rank	Publications		Co-Authored Publications (%)	Publications in English (%)	Average Number of Pages	WoS-IF Publications (%)	A + /A/B JOURQUAL Publications (%)	A + /A JOURQUAL Publications (%)	Name	Rank	Publications	Co-Authored Publications (%)	Publications in English (%)	Average Number of Pages	WoS-IF Publications (%)	A + /A/B JOURQUAL Publications (%)	A + /A JOURQUAL Publications (%)
P135	1	24.8		100	100	16.6	73	68	41	P135	1	22.0	100	100	16.6	73	68	41
P187	2	24.3		99	100	17.6	21	44	2	P123	2	19.8	95	100	15.5	82	82	25
P123	3	23.7		95	100	15.5	82	82	25	P187	3	19.1	99	100	17.6	21	44	2
P148	4	19.5		42	100	11.0	8	8	–	P093	4	16.1	43	100	7.0	–	–	–
P093	5	19.4		43	100	7.0	–	–	–	P143	5	15.5	86	100	17.4	67	31	28
P106	6	19.1		94	81	16.5	42	44	11	P146	6	15.3	98	100	18.6	40	31	13
P146	7	18.6		98	100	18.6	40	31	13	P021	7	14.8	94	100	15.5	58	50	39
P124	8	18.2		83	86	10.0	33	31	–	P171	8	13.2	96	100	16.4	11	9	–
P171	9	17.9		96	100	16.4	11	9	–	P106	9	12.9	94	81	16.5	42	44	11
P109	10	17.3		76	84	17.0	5	8	–	P033	10	12.2	95	100	17.4	12	59	46
P088	11	17.0		74	67	20.9	16	9	–	P133	11	12.2	87	100	17.7	77	71	26
P206	12	16.5		98	98	15.9	70	64	5	P136	12	11.5	100	100	20.0	71	69	38
P143	13	16.1		86	100	17.4	67	31	28	P206	13	10.9	98	98	15.9	70	64	5
P054	14	16.0		100	91	17.0	9	6	–	P229	14	10.8	100	100	18.8	89	73	24
P049	15	15.8		100	95	14.6	50	40	12	P049	15	10.0	100	95	14.6	50	40	12
P021	16	14.8		94	100	15.5	58	50	39	P213	16	10.0	97	94	9.6	22	25	–
P133	17	14.2		87	100	17.7	77	71	26	P226	17	9.9	100	93	17.1	83	73	7
P136	18	13.8		100	100	20.0	71	69	38	P022	18	9.4	97	100	14.5	33	33	25
P134	19	13.8		93	100	17.6	59	52	7	P052	19	9.4	91	100	13.2	87	65	13
P033	20	13.4		95	100	17.4	12	59	46	P178	20	9.2	86	95	12.0	32	41	18
P022	21	13.2		97	100	14.5	33	33	25	P225	21	9.1	100	100	17.6	87	87	9
P111	22	12.8		84	96	16.6	16	12	–	P032	22	8.9	100	100	18.0	15	58	48
P213	23	12.4		97	94	9.6	22	25	–	P019	23	8.4	80	100	21.0	70	50	40
Mean value per										Mean value per								
Leading Researcher (n = 23)		17.1		88	95	15.7	38	37	14	Leading Researcher (n = 23)		12.6	93	98	16.0	52	51	20
Non-Leading Researcher (n = 198)		3.9		86	72	15.9	30	30	10	Non-Leading Researcher (n = 198)		2.5	85	71	15.8	28	29	9
**V7 (Publication Lists, Fractional Counting, WoS-IF)**										**V8 (Publication Lists, Fractional Counting, A + /A/B JOURQUAL)**								
Name	Rank	Publication Points		Co-Authored Publications (%)	Publications in English (%)	Average Number of Pages	WoS-IF Publications (%)	A + /A/B JOURQUAL Publications (%)	A + /A JOURQUAL Publications (%)	Name	Rank	Publications	Co-Authored Publications (%)	Publications in English (%)	Average Number of Pages	WoS-IF Publications (%)	A + /A/B JOURQUAL Publications (%)	A + /A JOURQUAL Publications (%)
P123	1	74.8		95	100	15.5	82	82	25	P123	1	19.3	95	100	15.5	82	82	25
P171	2	73.6		96	100	16.4	11	9	–	P135	2	16.8	100	100	16.6	73	68	41
P135	3	54.6		100	100	16.6	73	68	41	P206	3	10.4	98	98	15.9	70	64	5
P093	4	47.9		43	100	7.0	–	–	–	P187	4	10.4	99	100	17.6	21	44	2
P187	5	46.6		99	100	17.6	21	44	2	P133	5	10.2	87	100	17.7	77	71	26
P106	6	37.7		94	81	16.5	42	44	11	P106	6	10.1	94	81	16.5	42	44	11
P229	7	33.0		100	100	18.8	89	73	24	P024	7	9.8	62	81	17.1	57	67	33
P206	8	32.1		98	98	15.9	70	64	5	P136	8	9.3	100	100	20.0	71	69	38
P052	9	31.9		91	100	13.2	87	65	13	P033	9	9.2	95	100	17.4	12	59	46
P033	10	29.6		95	100	17.4	12	59	46	P229	10	8.8	100	100	18.8	89	73	24
P133	11	29.1		87	100	17.7	77	71	26	P225	11	8.8	100	100	17.6	87	87	9
P049	12	29.0		100	95	14.6	50	40	12	P041	12	8.6	86	75	23.3	32	57	14
P146	13	28.2		98	100	18.6	40	31	13	P226	13	8.2	100	93	17.1	83	73	7
P226	14	27.0		100	93	17.1	83	73	7	P119	14	7.8	91	91	22.0	57	78	57
P136	15	26.9		100	100	20.0	71	69	38	P021	15	7.1	94	100	15.5	58	50	39
P225	16	26.6		100	100	17.6	87	87	9	P028	16	7.0	87	39	17.2	23	45	10
P127	17	26.4		93	100	17.5	79	71	64	P199	17	6.9	97	90	22.8	53	73	33
P209	18	26.1		100	100	12.8	100	88	88	P146	18	6.7	98	100	18.6	40	31	13
P178	19	26.0		86	95	12.0	32	41	18	P134	18	6.7	93	100	17.6	59	52	7
P199	20	24.8		97	90	22.8	53	73	33	P052	20	6.3	91	100	13.2	87	65	13
P217	21	23.0		100	100	16.7	94	75	75	P049	21	6.2	100	95	14.6	50	40	12
P143	22	22.8		86	100	17.4	67	31	28	P032	22	6.1	100	100	18.0	15	58	48
P053	23	22.6		100	85	16.1	61	44	24	P160	23	6.0	83	100	23.5	92	92	83
										P221	23	6.0	79	71	13.1	43	71	29
Mean value per										Mean value per								
Leading Researcher (n = 23)		34.8		94	97	16.3	60	57	26	Leading Researcher (n = 24)		9.0	93	93	18.0	58	63	26
Non-Leading Researcher (n = 198)		5.6		85	71	15.8	27	28	9	Non-Leading Researcher (n = 197)		1.5	85	72	15.6	27	27	9
**V9 (Publication Lists, Full Counting, A + /A JOURQUAL)**										**V10 (Publication Lists, Fractional Counting, A + /A JOURQUAL)**								
Name	Rank	Publications	Co-Authored Publications (%)		Publications in English (%)	Average Number of Pages	WoS-IF Publications (%)	A + /A/B JOURQUAL Publications (%)	A + /A JOURQUAL Publications (%)	Name	Rank	Publications	Co-Authored Publications (%)	Publications in English (%)	Average Number of Pages	WoS-IF Publications (%)	A + /A/B JOURQUAL Publications (%)	A + /A JOURQUAL Publications (%)
P135	1	34	100		100	16.6	73	68	41	P135	1	9.5	100	100	16.6	73	68	41
P033	2	19	95		100	17.4	12	59	46	P033	2	6.7	95	100	17.4	12	59	46
P136	3	17	100		100	20.0	71	69	38	P021	3	5.6	94	100	15.5	58	50	39
P032	4	16	100		100	18.0	15	58	48	P024	4	5.5	62	81	17.1	57	67	33
P123	5	14	95		100	15.5	82	82	25	P136	5	5.3	100	100	20.0	71	69	38
P021	5	14	94		100	15.5	58	50	39	P119	6	5.2	91	91	22.0	57	78	57
P209	5	14	100		100	12.8	100	88	88	P209	6	5.2	100	100	12.8	100	88	88
P119	8	13	91		91	22.0	57	78	57	P123	8	5.0	95	100	15.5	82	82	25
P217	9	12	100		100	16.7	94	75	75	P160	9	5.0	83	100	23.5	92	92	83
P053	10	10	100		85	16.1	61	44	24	P032	10	5.0	100	100	18.0	15	58	48
P143	10	10	86		100	17.4	67	31	28	P019	11	4.4	80	100	21.0	70	50	40
P199	10	10	97		90	22.8	53	73	33	P217	12	4.3	100	100	16.7	94	75	75
P219	10	10	100		90	14.6	85	80	50	P143	13	4.2	86	100	17.4	67	31	28
P160	10	10	83		100	23.5	92	92	83	P133	14	4.2	87	100	17.7	77	71	26
P229	15	9	100		100	18.8	89	73	24	P127	15	4.1	93	100	17.5	79	71	64
P022	15	9	97		100	14.5	33	33	25	P199	16	3.6	97	90	22.8	53	73	33
P218	15	9	93		93	15.6	14	71	64	P219	16	3.6	100	90	14.6	85	80	50
P127	15	9	93		100	17.5	79	71	64	P218	18	3.2	93	93	15.6	14	71	64
P133	19	8	87		100	17.7	77	71	26	P106	19	3.1	94	81	16.5	42	44	11
P019	19	8	80		100	21.0	70	50	40	P022	20	2.9	97	100	14.5	33	33	25
P106	21	7	94		81	16.5	42	44	11	P139	20	2.9	100	100	26.9	89	78	78
P049	21	7	100		95	14.6	50	40	12	P146	22	2.8	98	100	18.6	40	31	13
P024	21	7	62		81	17.1	57	67	33	P144	22	2.8	95	100	24.4	58	58	26
P169	21	7	82		91	26.7	73	91	64									
P139	21	7	100		100	26.9	89	78	78									
Mean value per										Mean value per								
Leading Researcher (n = 25)		12	93		96	18.2	64	65	45	Leading Researcher (n = 23)		4.5	93	97	18.4	62	64	45
Non-Leading Researcher (n = 196)		1	85		71	15.5	26	27	6	Non-Leading Researcher (n = 198)		0.4	85	71	15.5	27	27	7

**Fig 1:**
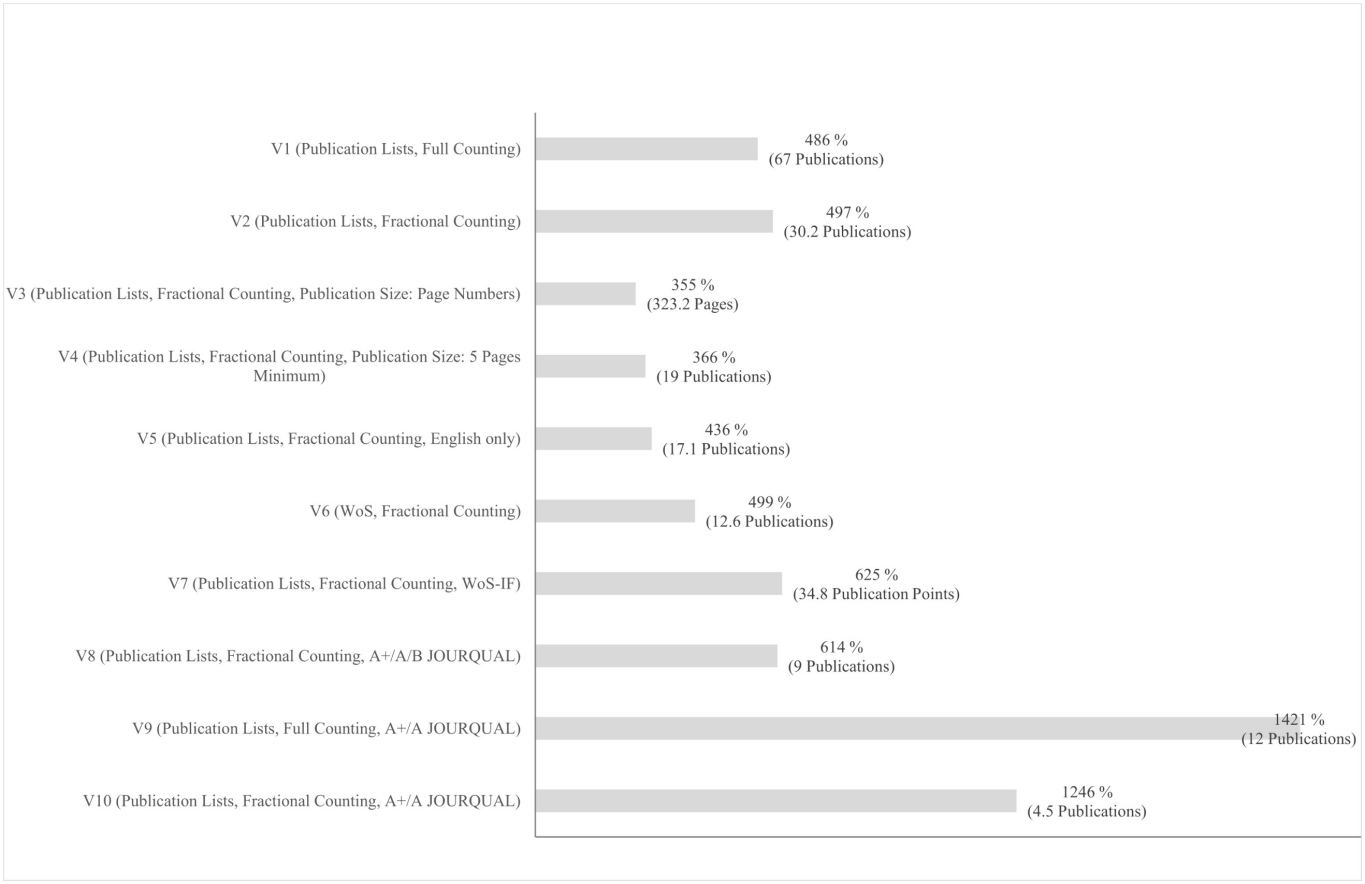
Performance advantage of leading researchers compared to non-leading researchers (Mean values for leading researchers).

A key element of our analysis is the final two rows of each ranking, which compare the average metrics of the leading researchers (’Mean value per Leading Researcher’) with those of the remaining group (’Mean value per Non-Leading Researcher.’ These values enable a clear distinction between the two cohorts. Fig 1 summarizes these comparisons and highlights the performance of the leading researchers, using the non-leading group as the reference point (100%).

As shown in Fig 1 and the final two rows of Table 5, the mean performance of leading researchers exceeds that of the non-leading group by a factor ranging from 1:3.55 (V3) to 1:14.21 (V9) across all ranking variants. The ratio 1:14.21 for V9, which considers publication lists, full counting, and A + /A JOURQUAL journals, illustrates that the leading researchers have, on average, fourteen times more publications in top-ranked journals than their peers. These results highlight the substantial performance gap between the two cohorts.

Notably, Category 1 rankings (V1–V5) show much more minor differences (ratios from 1:3.55 to 1:4.97) compared to Category 2 rankings (V6–V10), where the ratios range from 1:4.99 to 1:14.21.

### 3.2 Leading researchers: aggregate rankings

Table 6 identifies 63 researchers who achieved leading status in at least one of the ten rankings. Three appear as leading researchers in all variants, and nine in at least six. To be part of this elite group, a researcher must have achieved leading status in both Category 1 **and** Category 2 **rankings.**

**Table 6:** Leading researchers: aggregate ranking 1 (Categories 1 and 2).

Name	Aggregate	Leading in Category 1 Rankings					Leading in Category 1	Leading in Category 2 Rankings					Leading in Category 2	Leading in both categories
	Rank	V1	V2	V3	V4	V5	V1-V5	V6	V7	V8	V9	V10	V6-V10	V1-V10
P123	1	1	1	1	1	1	5	1	1	1	1	1	5	10
P106	1	1	1	1	1	1	5	1	1	1	1	1	5	10
P135	1	1	1	1	1	1	5	1	1	1	1	1	5	10
P146	4	1	1	1	1	1	5	1	1	1		1	4	9
P136	5	1		1		1	3	1	1	1	1	1	5	8
P187	5	1	1	1	1	1	5	1	1	1			3	8
P143	7			1	1	1	3	1	1		1	1	4	7
P171	7	1	1	1	1	1	5	1	1				2	7
P049	7	1			1	1	3	1	1	1	1		4	7
P033	10					1	1	1	1	1	1	1	5	6
P206	10	1			1	1	3	1	1	1			3	6
P133	10					1	1	1	1	1	1	1	5	6
P088	13	1	1	1	1	1	5							5
P021	13					1	1	1		1	1	1	4	5
P082	15	1	1	1	1		4							4
P090	15	1	1	1	1		4							4
P099	15	1	1	1	1		4							4
P086	15	1	1	1	1		4							4
P151	15	1	1	1	1		4							4
P032	15							1		1	1	1	4	4
P063	15	1	1	1	1		4							4
P199	15								1	1	1	1	4	4
P093	15		1			1	2	1	1				2	4
P054	15	1		1	1	1	4							4
P022	15					1	1	1			1	1	3	4
P229	15							1	1	1	1		4	4
P109	15		1	1	1	1	4							4
P081	15	1	1	1	1		4							4
P024	15				1		1			1	1	1	3	4
P217	30								1		1	1	3	3
P160	30									1	1	1	3	3
P225	30							1	1	1			3	3
P119	30									1	1	1	3	3
P226	30							1	1	1			3	3
P019	30							1			1	1	3	3
P127	30								1		1	1	3	3
P148	30		1		1	1	3							3
P209	30								1		1	1	3	3
P052	30							1	1	1			3	3
P124	30	1	1			1	3							3
P153	41	1	1				2							2
P041	41			1			1			1			1	2
P156	41	1	1				2							2
P053	41								1		1		2	2
P218	41										1	1	2	2
P213	41					1	1	1					1	2
P210	41	1	1				2							2
P219	41										1	1	2	2
P139	41										1	1	2	2
P028	41			1			1			1			1	2
P178	41							1	1				2	2
P113	41	1		1			2							2
P134	41					1	1			1			1	2
P065	54				1		1							1
P144	54											1	1	1
P043	54			1			1							1
P111	54					1	1							1
P233	54		1				1							1
P011	54				1		1							1
P112	54			1			1							1
P103	54		1				1							1
P221	54									1			1	1
P169	54										1		1	1

Among the remaining 51 leading researchers, a clear division emerges between the two categories. Notably, each of the eleven researchers who led in exactly three rankings did so exclusively within a single category, further validating our classification of ranking variants. As a result, our discussion will now focus on the five Category 2 rankings (V6–V10).

**Fig 2:**
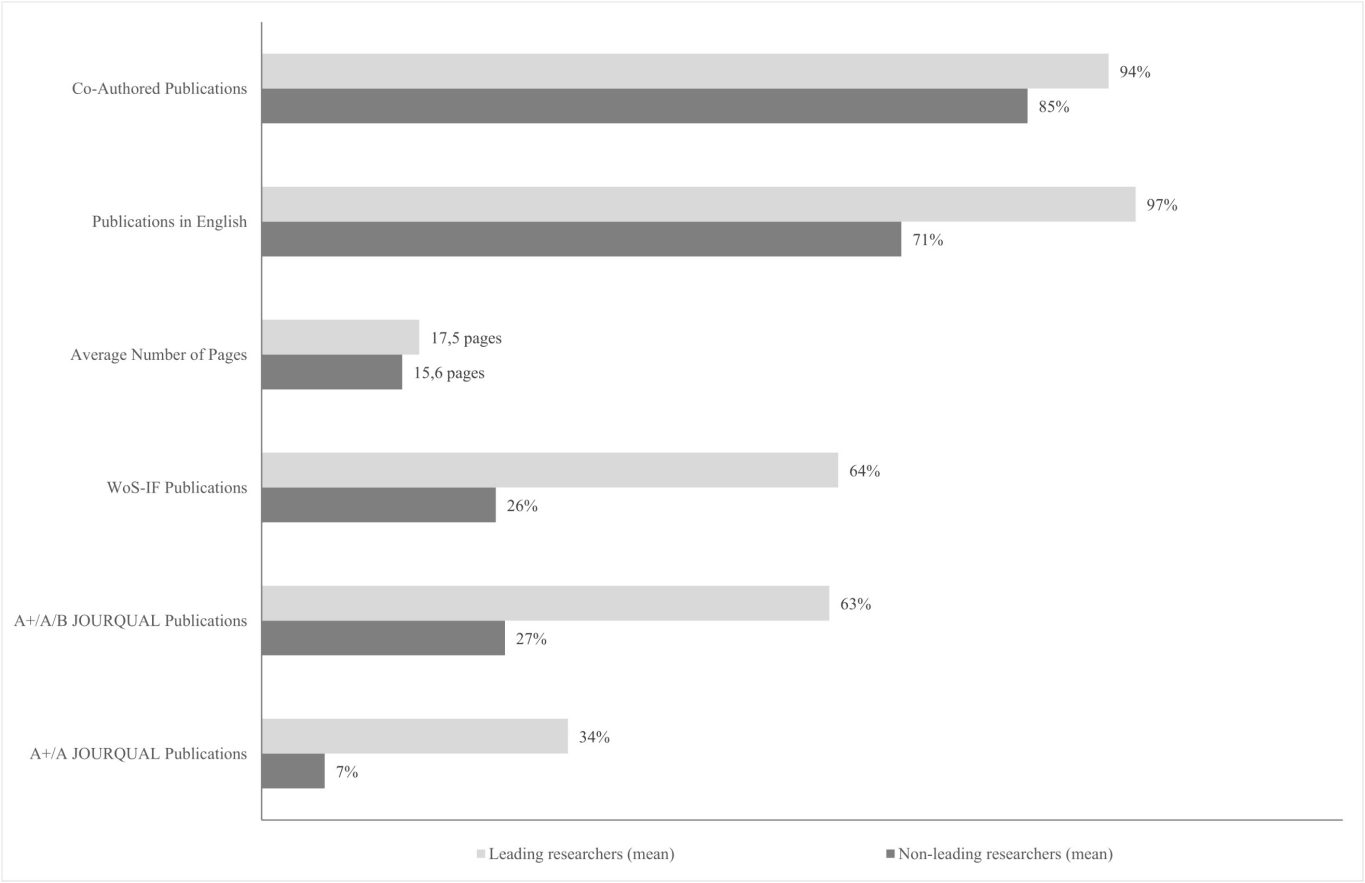
Research attributes of leading vs. non-leading researchers: aggregate ranking (Category 2).

Table 7 ranks 26 leading researchers based on their performance in Category 2 rankings (V6–V10). All appear among the leading researchers in at least three of these variants; six lead in all five. Comparing their performance to the non-leading cohort (see the last two rows of Table 7 and Fig. 2) reveals apparent differences. Leading researchers published more frequently in English, in WoS-indexed journals, and in A + /A/B JOURQUAL outlets. Specifically, 19 of the 26 scholars published exclusively in English, with an average share of 97%.

**Table 7:** Leading researchers: aggregate ranking 2 (Category 2).

Name	Category 2 Rank	Leading in Category 2 Rankings					Leading in Category 2	Co-Authored Publications (%)	Publications in English (%)	Average Number of Pages	WoS-IF Publications (%)	A + /A/B JOURQUAL Publications (%)	A + /A JOURQUAL Publications (%)
		V6	V7					V8	V9	V10	V6-V10
P123	1	1	1	1	1	1	5	95	100	15.5	82	82	25
P136	1	1	1	1	1	1	5	100	100	20.0	71	69	38
P106	1	1	1	1	1	1	5	94	81	16.5	42	44	11
P135	1	1	1	1	1	1	5	100	100	16.6	73	68	41
P033	1	1	1	1	1	1	5	95	100	17.4	12	59	46
P133	1	1	1	1	1	1	5	87	100	17.7	77	71	26
P143	7	1	1		1	1	4	86	100	17.4	67	31	28
P032	7	1		1	1	1	4	100	100	18.0	15	58	48
P021	7	1		1	1	1	4	94	100	15.5	58	50	39
P049	7	1	1	1	1		4	100	95	14.6	50	40	12
P199	7		1	1	1	1	4	97	90	22.8	53	73	33
P146	7	1	1	1		1	4	98	100	18.6	40	31	13
P229	7	1	1	1	1		4	100	100	18.8	89	73	24
P217	14		1		1	1	3	100	100	16.7	94	75	75
P160	14			1	1	1	3	83	100	23.5	92	92	83
P187	14	1	1	1			3	99	100	17.6	21	44	2
P225	14	1	1	1			3	100	100	17.6	87	87	9
P119	14			1	1	1	3	91	91	22.0	57	78	57
P022	14	1			1	1	3	97	100	14.5	33	33	25
P226	14	1	1	1			3	100	93	17.1	83	73	7
P019	14	1			1	1	3	80	100	21.0	70	50	40
P127	14		1		1	1	3	93	100	17.5	79	71	64
P206	14	1	1	1			3	98	98	15.9	70	64	5
P209	14		1		1	1	3	100	100	12.8	100	88	88
P052	14	1	1	1			3	91	100	13.2	87	65	13
P024	14			1	1	1	3	62	81	17.1	57	67	33
Mean value per													
Leading Researcher (n = 26)								94	97**	17.5*	64**	63**	34**
Non-Leading Researcher* (n = 195)								85	71**	15.6*	26**	27**	7**

**Note 1:** Table 5 includes only 221 of the 233 researchers, those who published at least one journal article during the study period. Asterisks (*) and (**) indicate statistically significant differences at the 5% and 1% levels, respectively.

**Note 2:** We used the two-sided Mann–Whitney U test for independent samples to assess the significance of differences between group means.

On average, 64% of their publications are WoS-indexed, and 63% appear in A + /A/B JOURQUAL journals, representing more than double the shares in the non-leading group (26% and 27%, respectively). For A**+** and A JOURQUAL publications, the contrast is even sharper: leading researchers average 34%, compared to just 7% in the non-leading cohort, nearly a fivefold difference.

## 4. Discussion

This study set out to address two key questions: first, how sensitive researcher rankings are to the choice of methodological specifications; and second, which publication attributes most strongly influence researchers’ positions within those rankings. The findings provide important insights for both research evaluation practice and the strategic considerations of early-career researchers.

### 4.1. Stability of leading positions across ranking variants

Our results demonstrate that Category 2 rankings, specifically, those that incorporate journal rankings or impact factors (V7–V10), consistently identify a relatively stable group of leading researchers. In contrast, the quantity-focused Category 1 rankings (V1–V5), which do not consider journal quality, produce more variation and instability in identifying leading performers (Fig. 1). This finding confirms earlier concerns raised in the literature that rankings based solely on publication counts are limited in their ability to identify consistent research excellence [50].

From a practical standpoint, these results support the prevailing approach in many academic appointment procedures, particularly in German-speaking countries, where quality-focused indicators, such as publications in highly ranked JOURQUAL or WoS-indexed journals, are prioritised. These measures not only better reflect the long-term academic impact of researchers but also align with studies suggesting that past high-quality productivity is a reliable predictor of future performance [51]. Hence, evaluation systems that rely heavily on quantity-based metrics risk overlooking those researchers who produce fewer but more impactful publications.

### 4.2. Publication attributes and their impact on rankings

Turning to our second research aim, the analysis of individual ranking variants reveals that specific publication attributes play a decisive role in determining a researcher's position.

Above all, journal quality emerges as the most influential factor. Leading researchers publish substantially more of their work in top-tier journals, with an average share of A + /A/B JOURQUAL publications more than twice that of their non-leading peers. This insight aligns with Mingers and Young (2016) [52], who emphasise the critical role of journal reputation in the business and management disciplines. These results also highlight the persistent tension between quality and quantity in academic publishing. The widespread ‘publish or perish’ culture has long incentivized quantity over quality, resulting in a flood of publications with few citations and reads [53]. However, critics question whether publishing in highly ranked journals is overrated, as these journals often prioritize methodological complexity over practical relevance [54]. Our results suggest that in quality-sensitive rankings, a small number of high-quality publications contributes more to a researcher's standing than a large volume of low-tier outputs, supporting recent calls to shift academic incentives away from volume-based metrics and toward impact- and reputation-sensitive measures [55].

Publication language also proves to be a strong differentiator. Leading researchers publish almost exclusively in English (ranging between 93% and 98% for rankings V5-V10), reflecting the international orientation of high-impact journals and evaluation systems. This fact has clear implications for early-career scholars: publishing in English is essential for global visibility and academic recognition. However, as Wei and Zhang (2020) [56] point out, the decline in national-language publications can create a disconnect between academic research and professional practice, particularly in non-English-speaking regions. A balanced strategy may therefore be warranted; one that prioritises English-language publications for career progression, while also maintaining contributions in national-language outlets to retain practitioner relevance.

Co-authorship, by contrast, does not significantly distinguish leading from non-leading researchers in our study. Nonetheless, it remains an important strategic factor. While full counting favours extensive co-authorship, fractional counting (used in most Category 2 rankings) eliminates its numerical advantage. Still, collaborations, particularly with experienced scholars, can offer early-career researchers essential learning opportunities and access to academic networks. As Xu and Pole [57] highlight, such networks are often crucial for building academic visibility and reputation, even when rankings may dilute individual credit.

Publication length, finally, appears to have a limited impact. It only influences rankings in variants where it is explicitly considered (V3 and V4). Even then, its practical relevance is minor, as most current evaluation systems do not include article length as a performance criterion (see Section 1.2). Early-career researchers are therefore better advised to focus on clarity, methodological rigour, and journal fit, rather than aiming for excessive length.

### 4.3. Strategic implications for early-career researchers

Taken together, the results provide several recommendations for early-career researchers seeking to establish a strong academic profile.

1. Publishing in English and journals indexed in WoS, carrying a high WoS-IF, and ranked highly in JOURQUAL is essential. For early-career researchers, however, focusing exclusively on quality is challenging. Publishing in top-tier journals often takes years and may not be successful at all. Early-career researchers must therefore carefully consider whether multiple publications in lower-tier or unranked journals will be viewed more favourably, particularly in future job applications, than time-consuming or unsuccessful attempts to publish in prestigious outlets. Moreover, regarding impact factors, early-career researchers must also consider that the WoS-IF changes annually, with the common 2-year WoS-IF fluctuating more than the 5-year WoS-IF. Ideally, rankings should use the WoS-IF as it stood at the time of publication; however, most studies, including ours, apply the WoS-IF current at the time of compiling the ranking. JOURQUAL ratings, however, remain stable for long periods, offering consistency but may lag behind recent shifts in journal influence. When in doubt, let JOURQUAL guide journal selection, as it comprehensively captures the top tier. Additionally, young researchers could also consult best-practice guidance on publishing in premier outlets (e.g., [58]). By targeting reputable, WoS‑indexed, and highly ranked journals, researchers maximize their visibility and competitiveness in evaluation procedures that heavily weight these metrics.
2. Collaborating strategically with experienced researchers can accelerate learning, extend networks, and enhance the quality and credibility of the work, provided that all co-authors make meaningful contributions [57]. Although full counting is increasingly regarded as outdated in research evaluation, placing greater pressure on early-career researchers to meet publication targets under fractional counting, they can still benefit greatly from collaboration. Joint work can support topic selection, broaden research perspectives, and help identify appropriate journals for publication.
3. Prioritizing clarity and rigour over article length and aligning submissions with the standards and expectations of high-impact journals remains critical. Given the limited importance of article length in evaluation practices and the growing emphasis on word limits in academic publishing, early-career researchers should identify a suitable outlet before writing. This procedure allows them to tailor the article's length and structure to the journal's specific requirements. Crucially, early-career researchers should be aware that evaluation practices increasingly favour journal quality and international visibility over sheer publication volume.

### 4.4. Limitations

Our study is not without limitations. Notably, university rankings, regardless of level, are often viewed critically or outright rejected by parts of the academic community [59]. While we acknowledge these concerns, our use of multiple ranking systems seeks to mitigate inherent biases.

Our decision to focus exclusively on publications in scientific journals merits critical reflection. This approach excludes other relevant forms of scholarly output, such as citations, different types of publications (e.g., monographs or conference papers), academic awards, or editorial roles, thereby potentially overlooking critical dimensions of academic performance. However, this limitation is grounded in the widely acknowledged centrality of journal publications within the field of business administration [16]. Expanding the scope to include additional output types would require reliance on individual publication lists, as international databases primarily index journal articles. However, this approach introduces a further challenge: the need to compare fundamentally different formats, such as a comprehensive monograph from a reputable publisher versus a brief, non-indexed journal article, which complicates the fair and consistent assessment of academic quality.

Despite incorporating ten different rankings from the literature, our aggregate rankings inevitably emphasized specific variants over others. This insight may prompt early-career researchers to reflect critically on the influence of particular ranking methodologies. Some variants rely on questionable criteria but were included due to their practical relevance. A prominent example is full counting of co-authored publications, which tends to overestimate individual contributions [60], especially as the number of co-authors increases. This problem is even more pronounced in citation analyses, where full counting remains the norm [15], despite a few exceptions [61].

We also considered publication language and length in our analysis, albeit with caution. Restricting the scope to specific languages does not inherently improve the validity of the results. Likewise, favoring longer publications risks prioritizing volume over quality. Nevertheless, setting a minimum length threshold can act as a quality proxy, as databases like WoS and JOURQUAL typically exclude short articles. Continued vigilance is required to monitor trends that may favor shorter formats.

Notwithstanding the general reservations about using the WoS-IF as a proxy for quality [62], we acknowledge alternative metrics such as SCImago Journal Rank and CiteScore. We justify the use of the WoS-IF with its frequent application in academic evaluations. Regarding subject-specific rankings, such as JOURQUAL [37], it is crucial to consider their subjective nature, in contrast to ostensibly rule-based indicators developed by commercial providers, which, however, may lack transparency. Despite the limitations of the WoS-IF and JOURQUAL, we believe they offer an adequate opportunity for assessing publication quality.

Due to the considerable effort involved in collecting data from personal publication lists, we limited our sample to experienced researchers at Austrian public universities. While this approach diverges from broader methods in the literature, it allowed us to reduce noise and focus on the qualitative nuances of performance measurement. We excluded early-career researchers (pre-docs and post-docs) intentionally to minimize bias in the interpretation of rankings and their implications.

Additionally, our analysis does not differentiate among the various sub-disciplines within business administration, partly due to the limited sample size. This approach may be problematic in light of diverse evaluation standards across different fields [22]. For instance, publishing in English may not be as advantageous in disciplines such as external accounting, where national legal frameworks prevail. However, for scholars pursuing international careers, a focus on globally relevant themes and English-language publications may be beneficial. While the study's findings may be transferable to other national contexts, applying them across disciplines would require adapting to field-specific publication standards and norms.

Finally, we want to emphasize that while our recommendations for early-career researchers may seem self-evident, even seemingly obvious strategies require empirical confirmation through systematic research.

## 5. Conclusions

Our study demonstrates that the specification of ranking criteria can substantially influence the research performance of individual scholars, confirming findings from earlier studies [22,43,63]. To be classified as a leading researcher in business administration, it is advisable to be familiar with the diversity and frequency of ranking variants applied in practice. However, accomplishing this is far from straightforward. While rankings commonly used in academic research can be identified through literature analyses, gaining insight into ranking procedures used in university hiring decisions, particularly academic appointments, remains far more challenging. Information on the actual use and design of such methods remains opaque, and the scholarly literature on this topic is limited.

Our study aimed to help close this information gap by drawing on existing academic sources as well as additional insights from appointment processes for senior academic positions. Classifying ranking approaches into quantity-oriented (Category 1) and predominantly quality-oriented (Category 2) variants supported our hypothesis that the latter are more relevant for evaluating research performance in business administration. Indeed, Category 2 rankings yielded substantially greater performance differentials between leading researchers and their peers, highlighting their discriminatory power and practical relevance. Based on these findings, we recommend that early-career researchers align their publication strategies with the logic of Category 2 rankings to strengthen their career prospects.

To facilitate practical guidance, we synthesised the results across all Category 2 variants to identify consistent traits associated with leading researchers. Most notably, placing articles in journals indexed by Web of Science (WoS) or in discipline-specific rankings, such as JOURQUAL (particularly relevant in German-speaking contexts), and fostering publication in English significantly enhances visibility and perceived quality. Given the considerable overlap between high-ranking JOURQUAL journals (A+ and A-) and the journals indexed in WoS, the use of WoS data alone is generally sufficient for performance evaluations. For highly ranked journals, time-consuming analyses of individual publication lists offer no advantage, as they are unlikely to yield additional relevant information when high-quality journal publications are the sole focus.

## Supporting information

S1 File Raw data. (7Z)
